# Novel characteristics of alpha-fetoprotein (AFP)-producing gastric cancer

**DOI:** 10.18632/oncotarget.22109

**Published:** 2017-10-30

**Authors:** Wenhui Sun, Baoqing Liu, Juntao Chen, Ping Gong, Xiaoying Wu, Chen Liu, De Zhou, Bo Hong, Weihua Gong

**Affiliations:** ^1^ Department of Surgery, Fuyang People's Hospital, Hangzhou City, People's Republic of China; ^2^ Department of Surgery and Medicine, Second Affiliated Hospital of School of Medicine, Zhejiang University, Hangzhou City, People's Republic of China; ^3^ Department of Oncology, The First Affiliated Hospital, Shihezi University School of Medicine, Shihezi City, People's Republic of China; ^4^ Tongde Hospital of Zhejiang Province, Hangzhou City, People's Republic of China; ^5^ Department of Hematology, First Affiliated Hospital of Medical School of Zhejiang University, Hangzhou City, People's Republic of China; ^6^ Department of Pathology, Second Affiliated Hospital of Medical School of Zhejiang University, Hangzhou City, People's Republic of China

**Keywords:** novelcharacteristics, AFP, non-AFP-producing, gastric cancer

## Abstract

**Background:**

Although serum AFP level is increased in the AFP-producing gastric cancer (GC) patient, AFP-producing GC may become recurrent without re-elevation of serum AFP level. Therefore, novel characteristics of AFP-producing GC such as other biomarkers are to be explored.

**Results:**

35 AFP-GC and 48 non-AFP-GC patients were included. Our present study revealed that blood type O (15 cases, 42.8%) and A (18 cases, 37.5%) were predominantly observed among AFP-GC and non-AFP-GC patients, respectively. Intriguingly, the least consistently was blood type AB (8.5%, 14.6%). Among AFP-GC patients, pre-operative serum AFP levels were weakly and reversely associated with pre-operative percentage of lymphocytes in the peripheral blood (R2 = 0.1556). The level of alkaline phosphatase (ALP) was highly correlated with the level of lactate dehydrogenase (LDH) (R^2^ = 0.6682) and CEA (R^2^ = 0.6813). The level of CEA was moderately correlated with the level of LDH (R^2^ = 0.3903). By contrast, no any associations between ALP, LDH, CEA, AFP, and peripheral lymphocytes were observed among those 48 non-AFP-GC patients.

**Materials and Methods:**

Retrospective analysis was carried out for 2773 inpatients of gastric cancer from January 2012 to November 2016 in the Second Affiliated Hospital of Zhejiang University School of Medicine.

**Conclusions:**

The aggressive biological features of AFP-producing GC are closely associated with abnormality of other tumor biomarkers and decrease of the proportion of peripheral lymphocytes, implying that post-operative increase of peripheral lymphocytes might benefit GC patients. Combined use of multiple biomarkers is of significance in comprehensive evaluation of AFP-producing GC patients and assessment for those with high risk of cancer recurrence.

## INTRODUCTION

Alpha-fetoprotein (AFP) was firstly detected in the human fetus in 1956, which is normally synthesized in the fetal liver as early as 6 weeks of gestation [1, 2]. Increase of serum AFP level one-year after birth is indicative of either hepatocellular carcinoma or yolk sac tumor [3]. Although the incidence of AFP-producing gastric cancer (GC) is not high (approximately 1.2–15%), its aggressive features and poor prognosis have recently acquired a lot of attention for further studies such asliver metastasis, vascular invasion, and lymph node metastasis [4]. Intensive exploration of optimal therapeutic strategies is required to achieve better patients’ survival [3]. In this present study, thirty-five cases of AFP-producing gastric cancer were retrospectively summarized and intriguingly a couple of characteristics of AFP-producing GC were identified.

Gastric cancer is one of the most common malignant tumors in the world. Its mortality rate occupies one fifth of all tumors [3]. It was reported that the diagnostic rate for early stage gastric cancer in China is as low (about 10%) as that in the western countries owing to opportunistic screening although endoscopy is widely available in rural and urban areas. Most gastric cancer was unfortunately diagnosed at advance stage [5, 6]. Most of GC cases in our study were in the advanced stage. Therefore, our research findings on AFP-producing GCs are of great value even for the western countries.

In the past, we systematically summarized biological behavior and molecular characteristics of AFP-producing GC [3]. Noticeably and importantly, serum AFP level is not well-studied for postoperative follow-up. It was reported that AFP-producing GC became recurrent without re-elevation of serum AFP level [7]. Therefore, our present study retrospectively analyzed thiry-five cases with elevated serum AFP levels. A couple of novel characteristics of AFP-producing GC were identified such as the relationships betweenalkaline phosphatase (ALP), lactate dehydrogenase (LDH), carcino-embryonic antigen (CEA), AFP, and percentage ofperipheral lymphocytes. These findings will shed light on further understanding AFP-producing gastric cancer and developing interventional therapeutics to improve prognosis in future clinical practice.

## RESULTS

### Characteristics of AFP-producing gastric cancer

2,773 cases of gastric cancers during 2012–2016 were found in our hospital's database. Of them, there is 35 AFP-GC patients (1.26%) at the age of 66.0 ± 10.9 years and their serum AFP level is 574.1 ± 1708.2 ng/mL. The ratio of male and female ratio is 1.5. 15 patients (42.8%) were blood type O positive, whereas the least is blood type AB-positive (8.5%). Metastases were detected in 25 (71.4%) AFP-producing GC patients, 5 (14.3%) liver metastasis and 20 (57.1%) metastasis of lymph nodes. Nevertheless, 28 (80%) patients received surgeries including palliative or radical surgeries. 12 cases of cardia (34.3%) and 7 cases of body (20%) of gastric adenocarcinoma were found, whereas 16 tumors were located in the antrum part (45.7%) (Table 1). The clinicopathological features showed that 90% of AFP-producing GC patients have T2 or deeper invasion. 34 (97.1%) patients underwent radical gastric cancer surgeries (Table 1). Furthermore, our findings revealed that high levels of peripheral AFP did not reflect the degree of tumor invasion in pathology. The patients with high levels of serum AFP (all > 250 ng/mL) might successfully receive radical gastrectomy. Pathological findings manifested that 34 (97.15%) cases were poorly differentiated adenocarcinoma (PDA) (22 cases, 62.85%) or moderately differentiated adenocarcinoma (MDA) (12 cases, 34.3%). Only one AFP-producing GC patient had well differentiated adenocarcinoma (WDA) (Table 1).

**Table 1:** Clinicopathologic features in the thirty-five AFP-producing patients

Patients		Value
Age	(mean age)	66.0 ± 10.9
Serum AFP level	(ng/mL)	574.1 ± 1708.2
Sex		
	male	21 (60%)
	female	14 (40%)
Blood type		
	O	15 (42.8%)
	AB	3 (8.5%)
	A	9 (25.7%)
	B	7 (20%)
Surgery		28 (80%)
	palliative	1 (2.9%)
	radical	27 (97.1%)
Metastases		
	liver	5 (14.3%)
	LN	20 (57.1%)
	negative	10 (28.6%)
Tumor position		
	antrum	16 (45.7%)
	body	7 (20%)
	cardiac	12 (34.3%)
Pathology		
	PDA	22 (62.85%)
	MDA	12 (34.3%)
	WDA	1 (2.85%)

LN, lymph nodes; PDA, poorly differentiated adenocarcinoma; MDA, moderately differentiated adenocarcinoma; WDA, well differentiated adenocarcinoma

### Characteristics of non-AFP-producing gastric cancer

48 non-AFP-GC patients (1.73%) at the age of 61.2 ± 10.8 years and their serum AFP level is 2.9 ± 1.6 ng/mL. The ratio of male and female ratio is 1.53. 18 patients (37.5%) were blood type A positive, whereas the least is blood type AB-positive (14.6%). Only lymph nodes metastasis 32 (66.7%) was detected among non-AFP-producing GC patients. Therefore, all patients were received radical surgeries. 14 cases of cardia (29.2%) and 24 cases of body (50%) of gastric adenocarcinoma were found, whereas 10 tumors were located in the antrum part (20.8%). Pathological findings manifested that 43 (89.6%) cases were poorly differentiated adenocarcinoma (PDA) (23 cases, 47.9%) or moderately differentiated adenocarcinoma (MDA) (20 cases, 41.7%). Five (10.4%) non-AFP-producing GC patients had well differentiated adenocarcinoma (WDA) (Table 2).

**Table 2:** Clinicopathologic features in the forty-eight non-AFP-producing patients

Patients		Value
Age	(mean age)	61.2 ± 10.8
Serum AFP level	(ng/mL)	2.9 ± 1.6
Sex		
	male	48 (60.4%)
	female	19 (39.6%)
Blood type		
	O	11 (22.9%)
	AB	7 (14.6%)
	A	18 (37.5%)
	B	12 (25%)
Surgery		28 (80%)
	palliative	0 (0)
	radical	48 (100%)
Metastases		
	liver	0 (0)
	LN	32 (66.7%)
	negative	16 (33.3%)
Tumor position		
	antrum	10 (20.8%)
	body	24 (50%)
	cardiac	14 (29.2%)
Pathology		
	PDA	23 (47.9%)
	MDA	20 (41.7%)
	WDA	5 (10.4%)

LN, lymph nodes; PDA, poorly differentiated adenocarcinoma; MDA, moderately differentiated adenocarcinoma; WDA, well differentiated adenocarcinoma.

### Novel characteristics of biomarkers among these AFP-producingGC patients in comparison with non-AFP-producing GC patients

**Figure 1:**
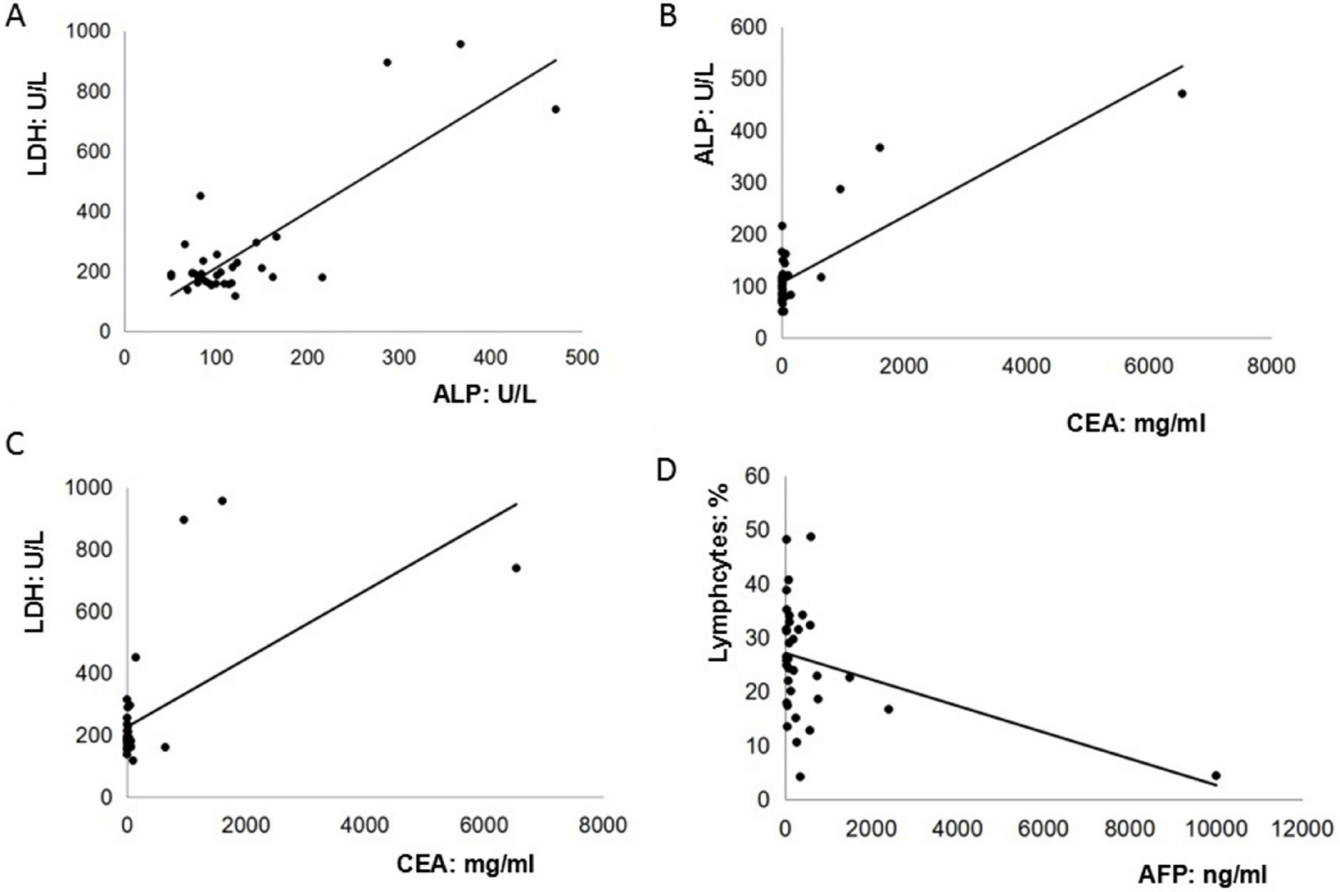
The relationships among peripheral biomarkers in these thirty-five AFP-producing patients (**A**). The level of alkaline phosphatase (ALP) was highly correlated with the level of lactate dehydrogenase (LDH) (R^2^ = 0.6682); (**B**). The level of CEA was highly correlated with the level of ALP (R^2^ = 0.6813); (**C**). The level of CEA was moderately correlated with the level of LDH (R^2^ = 0.3903); (**D**). The level of AFP was weakly and reversely correlated with the percentage of peripheral lymphocytes (R^2^ = 0.1556).

**Figure 2:**
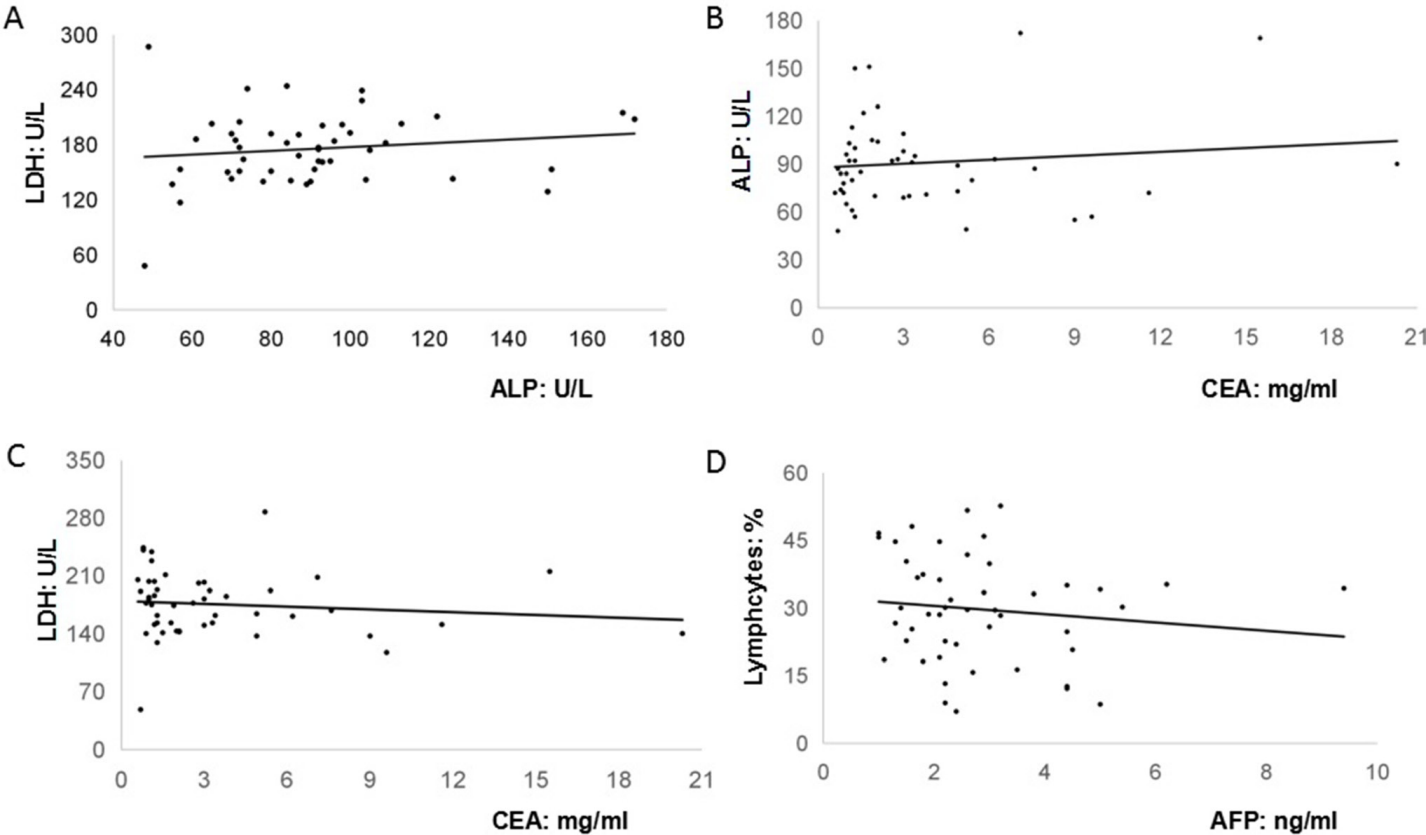
The relationships among peripheral biomarkers in these forty-eight non-AFP-producing patients (**A**). The level of alkaline phosphatase (ALP) was not significantly associated with the level of lactate dehydrogenase (LDH) (R^2^ = 0.021); (**B**). The level of CEA was not significantly associated with the level of ALP (R^2^ = 0.0141); (**C**). The level of CEA was not significantly associated with the level of LDH (R^2^ = 0.013); (**D**). The level of AFP was not significantly associated with the percentage of peripheral lymphocytes (R^2^ = 0.0152).

Although significant pattern was not detected in the cancer antigen 19–9 (CA19–9), carcino-embryionic antigen (CEA) and AFP levels among GC patients with both negative and positive cytology serum and peritoneal lavage fluid [9], it was found that anemia and elevated levels of serum CEA, CA19-9, CA125 were frequently detected among 80% (28/35), 60% (21/35), 42.9% (15/35), 20% (7/35) of AFP-producing GC patients, particularly for those with serosal involvement, lymphatic and venous invasion, and lymph node metastasis [10]. In addition, our findings exhibited that there were 18 cases (51.4%) with low serum level of albumin (Total: 3.5 ± 0.4 g/dL). Therefore, our present study attempted to analyze the relationships among peripheral biomarkers in these 35 AFP-producing patients. Our present study firstly revealed that pre-operative serum AFP levels were not associated with other biomarkers such as CA125, CEA or CA 19–9. However, pre-operative serum AFP levels were weakly and reversely associated with pre-operative percentage of lymphocytes in the peripheral blood (R^2^ = 0.1556). Furthermore, it was interestingly found that the level of alkaline phosphatase (ALP) was highly correlated with the level of lactate dehydrogenase (LDH) (R^2^ = 0.6682) and CEA (R^2^ = 0.6813). The level of CEA was moderately correlated with the level of LDH (R^2^ = 0.3903) (Figure 1). By contrast, no any associations between ALP, LDH, CEA, AFP, and peripheral lymphocytes were observed among those 48 non-AFP-GC patients (ALP and LDH, R^2^ = 0.021; CEA and ALP, R^2^ = 0.0141; CEA and LDH, R^2^ = 0.013; AFP and peripheral lymphocytes, R^2^ = 0.0152) (Figure 2).

## DISCUSSION

Sano T et al. reported that AFP-producing tumors occupied 1.6% among GC patients. Of them, 79% were nodal metastasis and 53% were liver metastasis. Preoperative serum AFP levels were not associated with tumor size, disease stage, depth of invasion or survival [11]. Herein, our study displayed relatively lower incidences of AFP-producing GC (1.26%), nodal metastasis (57.1%), and liver metastasis (14.3%). Pre-operative serum AFP levels could not represent any tumors’ characteristics such as tumor size, disease stage, and pathological status. Furthermore, synchronous or metachronous features were observed in clinic based on a relative large-scale study [4]. Thereafter, novel characteristics of AFP-producing GC were prospectively explored and analyzed.

In recent years the incidence of gastric cancer has decreased with development of diagnostic and therapeutic techniques including various biomarkers etc. Therefore, it is of great significance in studying convenient, economical, and noninvasive biomarkers. It is known that most commonly used biomarkers (e.g., CA19–9 and CEA)were closely associated with gastric cancer [12]. Augmented levels of CA19–9 and CEA were statistically associated with the 5-year disease free survival and overall survivalrates. CA 19–9 became an independent prognostic factor [10]. Our present study firstly revealed that pre-operative serum AFP levels were weakly associated with pre-operative percentage of lymphocytes in the peripheral blood in spite of no asscociation between pre-operative serum AFP levels and other biomarkers such as CA125, CEA or CA 19–9. Furthermore, anemia and elevated levels of serum CEA, CA19–9 were frequently detected among 80% (28/35), 60% (21/35), 42.9% (15/35) of AFP-producing GC patients, particularly for those with serosal involvement, lymphatic and venous invasion, and lymph node metastasis. Interestingly, it was found that pre-operative serum AFP levels were weakly associated with pre-operative percentage of lymphocytes in the peripheral blood, implying that suppression of systematic immune system might be involved in the progress of AFP-producing GC. Furthermore, our findings revealed that the level of alkaline phosphatase (ALP) was highly correlated with the level of both LDH and CEA. The level of CEA was moderately correlated with the level of LDH. These aforementioned data may in large measure hint or explain for the aggressive biological behaviors of AFP-producing GC such as a high incidence of venous invasion, lymphatic invasion, and metachronous and synchronous liver metastasis [3]. Further studies are required to unveil the underlying mechanisms in the future.

With respect to those patients with serum AFP greater than 300 ng/mL, pronouncedly poorer 1-, 3-, and 5-year survival rates were observed [3]. However, in our present study thirteen cases with serum AFP greater than 250 ng/mL successfully received radical gastrectomy surgeries accounting for their clinical stages. Therefore, earlier detection by using endoscopy and biomarkers is empirically required to improve the diagnosis and therapies for AFP-producing GC.

In addition, past studies had shown that ABO genotype was closely related with gastric cancer development [13–15]. Presence of the B allele indicated a significant lower risk of noncardia GC [13]. Our study exhibited a consistent finding on non-AFP-GC patients, which is a significant association between blood type A and gastric cancer [14]. Although decreased risk of gastric cancer was found in blood type O positive among common GC patients by a meta-analysis [15], our present study firstly unveil that blood type O (15 cases, 42.8%) positive were predominantly observed among AFP-GC patients.

We observed the stage of gastric cancer and percentage of T-cell [16]. The elevated pre-operative level of lymphocyte to monocyte ratio was a significant beneficial factor in the prognosis of stage II/III common GC patients [17]. Helicobacter pylori eradication might prevent DNA damage and oxidative stress in the peripheral lymphocytes, which decreases risk of gastric cancer development [18]. All aforementioned information supported a notion that peripheral lymphocytes played an important role in GC development. Higher percentage and avoidance of DNA damage of peripheral lymphocytes would reduce risk of gastric cancer. It is in line with our present findings, which showed higher percentages of peripheral lymphocytes among AFP-producing GC patients with lower AFP levels.

### Limitations of our present study

The rate of detection of early gastric cancer is relatively low in China, which is similarly observed in the western countries. Most of AFP-producing GC cases cannot be subject to gastrectomy surgeries. Therefore, our present study on these limited AFP-producing cases is not sufficient to prove the subsequent issues. For instance, large data on more cases are required to extrapolate our findings such as the correlations among aforementioned biomarkers. A rise of postoperative serum AFP level normally indicates tumor recurrence, whereas normal serum AFP level cannot exclude recurrent tumor [11]. It is undefined well whether pre- or post-operative peripheral AFP levels are positively associated with patients’ prognosis and survival. Although past studies exhibited various post-operative adjuvant chemotherapy regimens such as 5-fluorouracil, irinotecan, cisplatin, docetaxel, methotrexate, paclitaxel, mitomycin C or tegafur/uracil, no data displayed their advantages and differential survivals [11]. Optimal chemical regimens cannot be analyzed for AFP-producing GC particularly for those cases with high levels of peripheral AFP. Furthermore, H. Pylori infection was not detected in each patients and this data are missing in our present study. Therefore, the relationship between H. Pylori infection and AFP-producing GC could not be studied. All these research issues require more international collaborations and further investigations.

## MATERIALS AND METHODS

### Patients

From January 2012 to November 2016, 2,773 patients were searched in our database and primary gastric adenocarcinoma was diagnosed by histology at the Department of Surgery in the Second Affiliated Hospital of Zhejiang University School of Medicine. Preoperative conventional serum tumor biomarkers were tested including AFP CA19-9, CEA, CA125 in all inpatients. Among these GC patients, thirty-five had an elevated serum level of AFP (> 20 ng/mL).Forty-eight non-AFP GC patients were randomly selected as control group. Their serum level of AFP was lower than 20 ng/mL.Patients with primary hepatic diseases were excluded from our analysis. All these patients routinely received abdominal ultrasound, endoscopic examination, and computed tomographic scan to comprehensively evaluate the tumor parameters (size, location, and invasion depth) as well as the status of liver and lymph node metastasis. Staging was determined according to the American Joint Committee on Cancer (AJCC) TNM Staging Classification for Carcinoma of the Stomach (Seventh Edition, 2010) [8]. Data were achieved from patients’ pre-/post-operative and pathological reports. The raw data is publicly available upon request except patients’confidentiality.

### Serum assays for AFP and CEA

After these patients’ admission to our hospital, all patients underwent complete standard blood examinations, including blood cells count, lactic dehydrogenase (LDH), cholinesterase (ChE), alkaline phosphatase (ALP), and albumin. The sampled blood was centrifuged at 1,000g for 10 min in order to isolate the plasma from the blood cells. AFP, CEA, CA125, and CA19-9 were assayed by using magnetic particle enzyme immunoassay in immunoassay system (Beckman Coulter Inc., Fullerton, CA).All blood tests were performed according to the standard testing procedure by using the manufacturer's specified testing reagents inthe clinical laboratory.The cut-off value for serum AFP, CEA, CA125, and CA19-9were 20 ng/mL, 5 ng/mL, 35 U/mL, 37 U/mL, respectively, according to the manufacturer's instructions.

### Statistical analysis

The collected data were processed by the GraphPad Prism 5 software. Correlation analysis and student's *T*-test were performed for the analysis of data. Demographic data were analyzed by descriptive statistics. All the tests were two-sided and statistical difference was accepted as *p* < 0.05.

## CONCLUSIONS

The aggressive biological features of AFP-producing GC are closely associated with abnormality of other tumor biomarkers and decrease of the proportion of peripheral lymphocytes, implying that post-operative increase of peripheral lymphocytes might benefit GC patients. Combined use of multiple biomarkers is of significance in comprehensive evaluation of AFP-producing GC patients and assessment for those with high risk of cancer recurrence.

### Ethics approval and consent to participate

All authors confirm that our research complies with national guidelines for retrospective studies.

### Consent for publication

All co-authors consent to publish this manuscript.

### Availability of data and material

Not applicable.

## Abbreviations

AFP: alpha-fetoprotein
ALP: alkaline phosphatase
CA125: carbohydrate antigen
CA19–9: carbohydrate antigen
CEA: carcino-embryonic antigen
ChE: cholinesterase
GC: gastric cancer
LDH: lactate dehydrogenase
PDA: poorly differentiated adenocarcinoma
MDA: moderately differentiated adenocarcinoma
WDA: well differentiated adenocarcinoma
